# Anterior Odontoid Screw Fixation for Trauma: Case Series and Technical Considerations

**DOI:** 10.3390/jcm14217754

**Published:** 2025-10-31

**Authors:** Federica Figà, Marcello Nunzio Tirendi, Andrea Talacchi, Alessandro Olivi

**Affiliations:** 1 Institute of Neurosurgery, Fondazione Policlinico Universitario A. Gemelli IRCCS, Università Cattolica del Sacro Cuore, Largo Agostino Gemelli 8, 00168 Rome, Italy; 2 Department of Neurosurgery, San Giovanni-Addolorata Hospital, 00184 Rome, Italy

**Keywords:** anterior odontoid screw fixation, atlantoaxial instability, odontoid fracture

## Abstract

**Background/Objectives**: Odontoid fractures—prevalently Anderson–D’Alonzo type II—are clinically relevant for their biomechanical instability and risk of non-union. Posterior C1–C2 fusion yields the highest fusion rates but sacrifices atlantoaxial rotation. Anterior odontoid screw fixation (AOSF) enables direct osteosynthesis while preserving motion. This study aimed to evaluate the radiographic outcomes, fusion rate, and technical considerations of AOSF in a consecutive single-center series, highlighting anatomical and procedural factors influencing bone healing. **Methods**: Retrospective, single-center case series of patients who underwent AOSF for acute type II odontoid fractures (2018–2024). Inclusion criteria included CT-confirmed fractures with reducible alignment. Radiographic parameters (fracture gap and angulation) were measured on standardized sagittal CT reconstructions. Outcomes were evaluated at 6 weeks, 3 months, and 6 months. Mean follow-up was 24 months. **Results**: The mean fracture gap decreased from 5.3 mm preoperatively to 0.8 mm postoperatively, and angulation from 27.8° to 3.5° (*p* < 0.0001). Nine of ten patients (90%) achieved solid fusion; one required secondary posterior fixation. No intra- or postoperative infections, neurovascular injuries, or neurological deficits were observed. **Conclusions**: AOSF is a safe and effective motion-preserving technique in appropriately selected Grauer IIA/IIB fractures. Precise anatomical reduction (<2 mm gap, <5–10° angulation) is a key predictor of successful fusion, even in elderly patients. Future multicenter studies with larger cohorts and standardized clinical outcome measures are needed to validate radiographic thresholds and optimize patient selection.

## 1. Introduction

Fractures of the odontoid process, particularly type II lesions as defined by Anderson and D’Alonzo, represent a major subset of upper cervical trauma. The odontoid (dens) acts as the axial pivot between C1 and C2, accounting for ~50% of cervical axial rotation. Type II fractures are associated with a high risk of nonunion due to poor vascular supply and persistent biomechanical shear stress at the fracture interface [1].

Approximately 10–20% of all cervical spine injuries involve odontoid fractures, which are most common in the elderly following low-energy trauma such as ground-level falls [2]. Their incidence is expected to increase with aging populations and longer life expectancy.

The management of type II odontoid fractures is still subject to discussion. Conservative treatment with rigid cervical collars or halo-vest immobilization has traditionally been proposed as the first line of treatment for nondisplaced fractures. Nevertheless, high rates of fibrous nonunion and complications from prolonged immobilization have led many centers to give way to surgical stabilization.

Two principal surgical options exist: anterior odontoid screw fixation (AOSF) and posterior C1–C2 fusion. AOSF, as introduced by Böhler and later refined by Apfelbaum, enables direct osteosynthesis across the fracture line and preserves atlantoaxial motion. The technique is biomechanically preferable in cases with proper alignment and healthy transverse ligament, offering union rates between 85% and 95%.

However anatomical limitations such as severe kyphosis, comminution of the odontoid base, or an unfavorable fracture line angle may render the anterior approach unfavorable. In such cases, posterior fusion techniques—such as transarticular screw fixation (Magerl) or segmental fixation (Goel–Harms constructs)—provide excellent biomechanical stability but eliminate atlantoaxial rotation [3].

The Grauer classification refines the original Anderson–D’Alonzo system by incorporating fracture orientation and reducibility, helping guide treatment selection. Grauer type IIB fractures—horizontal fracture lines with reducible alignment—are particularly suited for AOSF [4].

Despite technical advances, the choice between conservative and surgical approaches continues to be influenced by patient age, comorbidities, bone quality, and surgeon experience.

Recent studies highlight evolving trends in odontoid fracture management, with a gradual shift toward tailored decision-making based on fracture morphology and patient-specific risk factors [5,6,7]. Moreover, minimally invasive approaches, including endoscopic-assisted anterior screw fixation, are emerging but remain limited to select centers [8].

In recent years, minimally invasive and percutaneous adaptations of anterior odontoid screw fixation have been described to streamline exposure and reduce soft-tissue morbidity while preserving the biomechanical advantages of direct osteosynthesis.

Among these, Umana and Visocchi et al. reported a percutaneous anterior odontoid screw technique that proved both safe and reproducible, achieving excellent alignment and fusion rates with minimal complications [9].

In parallel, growing evidence indicates that the timing of surgery is a relevant determinant of bone healing, with earlier fixation associated with higher fusion rates in appropriately selected patients [10].

This study aims to evaluate radiographic outcomes, fusion rates, and technical considerations of AOSF for acute type II odontoid fractures (Grauer IIA/IIB) in a consecutive single-center series. We hypothesize that accurate anatomical reduction is the primary determinant of successful bone union and that specific radiographic thresholds may predict outcomes.

## 2. Materials and Methods

### 2.1. Study Design

Our series derives from a retrospective analysis of 10 consecutively treated patients for the period from 2018 to 2024. Ethical review and approval were waived for this study because it involved only the retrospective analysis of fully anonymized clinical and radiological data collected as part of routine care, without any intervention, modification of treatment, or use of identifiable patient information, in accordance with the Declaration of Helsinki. Demographic data of the patients and clinical characteristics of their injuries are presented in Table 1.

### 2.2. Eligibility

Inclusion criteria:

- Diagnosis of acute Anderson and D’Alonzo type II odontoid fracture confirmed by high-resolution CT (slice thickness ≤ 1.25 mm) [11];
- Grauer type IIB or IIA pattern (horizontal plane, reducible alignment) [4];
- Time from injury ≤14 days, in accordance with evidence suggesting that delayed fixation beyond the second postoperative week may reduce fusion likelihood [10];
- Fracture reducible under dynamic radiography or fluoroscopic traction.

Exclusion criteria:

- Comminuted or posteriorly displaced fractures (Grauer IIC);
- Transverse ligament disruption or C1–C2 instability on MRI;
- Cervical kyphosis precluding anterior access;
- Severe osteoporosis or comorbidities contraindicating general anesthesia.

All patients were evaluated with dynamic X-rays and/or intraoperative traction to confirm alignment feasibility for anterior screw placement. All procedures were performed by a single surgeon (M.N.T.).

### 2.3. Imaging and Preoperative Planning

Preoperative imaging included cervical CT and MRI. CT angiography was additionally performed in selected cases when fracture extension close to the transverse foramen raised concern for vertebral artery (VA) proximity and its relationship to the C2 isthmus [12]. Fracture line orientation was measured; those with an angle ≤ 60° relative to the C2 endplate were considered suitable for anterior fixation [3]. Fracture gap and angulation were assessed on sagittal multiplanar reconstructions of high-resolution CT scans (slice thickness ≤ 1.25 mm), using integrated digital tools within the institutional PACS system (MedDream PACS, Softneta, Vilnius, Lithuania).

The fracture gap was defined as the minimum distance between the proximal and distal cortices at the fracture interface, measured orthogonally to the longitudinal axis of C2. Angulation was defined as the angle formed between the longitudinal axis of the odontoid process and that of the C2 vertebral body.

Measurements were obtained on standardized sagittal planes by two independent observers. Minor discrepancies were resolved by consensus, and inter-observer variability was assessed qualitatively to ensure consistency.

### 2.4. Surgical Technique

All surgeries were performed under general balanced anesthesia with the patient in the supine position on a radiolucent operating table and the head gently extended to facilitate exposure. A left-sided anterolateral transcervical approach was performed, following the principles of the classic anterior cervical approach described by Smith and Robinson [13], exposing the C2–C3 disc space under continuous biplanar fluoroscopic guidance. The carotid sheath was retracted laterally, while the tracheoesophageal complex was mobilized medially. Exposure was maintained using a Caspar cervical retractor system.

A guide wire was introduced from the anteroinferior aspect of the C2 vertebral body toward the odontoid apex, maintaining a sagittal insertion angle of 25–30° and a coronal deviation < 5° from the midline. The planned trajectory was verified in both anteroposterior and lateral fluoroscopic views. Cannulated drilling and tapping were performed over the guide wire, followed by insertion of a cannulated, partially threaded, self-tapping lag screw of appropriate dimensions (length 34–42 mm) for the individual patient. Interfragmentary compression was achieved under continuous fluoroscopic control.

The surgical site was irrigated, meticulous hemostasis ensured, and closure performed in anatomical layers. A subfascial drain was placed when indicated and removed within 24–48 h. Postoperative cervical immobilization was selectively applied based on intraoperative stability and bone quality.

To decrease intraoperative risks, several precautions were systematically adopted:

- Guidewire control: continuous fluoroscopic monitoring to prevent perforation through the odontoid tip or anterior C1 arch;
- Midline trajectory: use of AP fluoroscopic guidance and preoperative templating to avoid screw deviation;
- Esophageal and recurrent laryngeal nerve protection: careful blunt dissection and medial retraction of the tracheoesophageal complex;
- Vertebral artery safety: preoperative CT angiography and cautious drilling near the transverse foramen.

Single-screw fixation was generally preferred; a second screw may be considered in the presence of marked fracture obliquity or instability, although no second screws were used in this cohort, in line with prior evidence showing no clear advantage over one-screw fixation [14].

### 2.5. Postoperative Protocol

All patients underwent non-contrast cervical CT within 24 h from the surgical procedure to confirm implant positioning and fracture reduction. Mobilization typically began on postoperative day 2. A semi-rigid cervical collar was applied for 4 weeks in patients with osteoporotic bone or suboptimal intraoperative stability; others were managed without immobilization. Clinical and radiological follow-up was scheduled at 6 weeks, 3 months, and 6 months. Bone union was assessed on CT at 3 months.

### 2.6. Outcome Measures

The primary outcome was radiographic fusion rate at 3 months. Secondary outcomes included changes in fracture gap and angulation, measured pre- and postoperatively. Clinical outcomes such as VAS, NDI, or JOA scores were not included due to the retrospective nature and small sample size of this series. This limitation is acknowledged and justified by the study’s focus on radiographic predictors of union rather than functional outcomes, which require prospective validation in larger cohorts.

### 2.7. Statistical Analysis

Continuous variables were expressed as mean ± standard deviation (SD) and range. Pre- and postoperative values (fracture gap and angulation) were compared using paired *t*-tests (two-sided; significance threshold α = 0.05). Normality of the differences was assessed with the Shapiro–Wilk test (*p* > 0.05 in all cases) and visual inspection of Q–Q plots. Statistical analyses were performed using R version 4.3.2 (R Foundation for Statistical Computing, Vienna, Austria).

## 3. Results

A total of 10 patients (5 male, 5 female) with acute type II odontoid fractures underwent single-screw anterior odontoid screw fixation (AOSF). The median age was 76 years (range, 17–85), while the mean was 69.9 ± 19.6 years; no cases required placement of a second screw. Most injuries occurred following low- to moderate-energy trauma. Three patients had associated fractures: Humerus (1), Nasal bones (1), Radius (1).

The comparison of preoperative and postoperative displacement and angulation is summarized in Table 2.

Radiographic assessment showed a mean preoperative displacement of 5.27 ± 2.23 mm (range, 2.0–10.0 mm), which decreased to 0.77 ± 1.35 mm (range, 0–3.7 mm) postoperatively. A paired *t*-test demonstrated that this reduction was statistically significant (t = 11.8, df = 9, *p* < 0.0001). The mean angulation was reduced from 27.8° preoperatively to 3.5° postoperatively (t = 5.2, df = 9, *p* < 0.0005).

Bone consolidation occurred in 9 of 10 patients (90%); 1 patient developed non-union requiring secondary posterior fixation at 4 months. The patient who required revision had the largest residual postoperative gap (3.7 mm). No intraoperative complications were observed. Specifically, no cases of vertebral artery injury, esophageal injury, guidewire perforation, or screw malposition occurred. Postoperatively, there were no wound infections, dysphagia, or neurological deficits.

**Figure 1:**
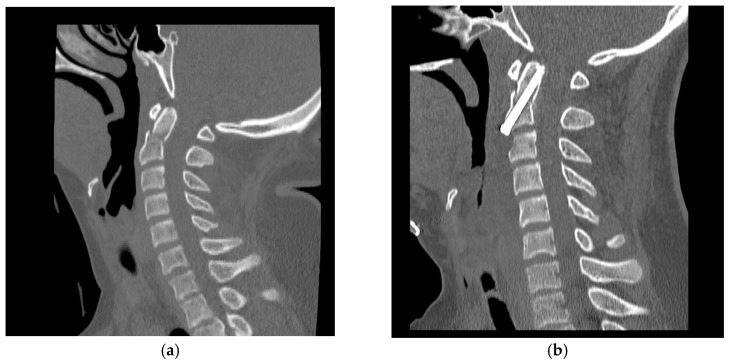
Sagittal CT scans of the cervical spine. (**a**) Preoperative image showing a type II odontoid fracture with displacement and angulation. (**b**) Postoperative image demonstrating correct placement of the anterior odontoid screw with anatomical reduction and restoration of alignment. Example from a 17-year-old male patient.

Cervical CT confirmed proper implant positioning and reduction within 24 h in all patients. This is illustrated in Figure 1, which shows preoperative displacement and postoperative alignment restoration. Dynamic radiographs and CT were repeated at 6 weeks, 3 months, and 6 months, with bone fusion primarily documented at 3 months.

Follow-up ranged from 12 to 36 months (median: 24 months). Individual follow-up durations were as follows: 12 months (2 patients), 18 months (3 patients), 24 months (3 patients), and 36 months (2 patients).

## 4. Discussion

This retrospective case series analyzed radiographic and clinical outcomes of anterior odontoid screw fixation (AOSF) for Anderson and D’Alonzo type II odontoid fractures. Solid bone fusion was achieved in 90% of patients, with a single case of non-union requiring posterior revision. Radiographic analysis demonstrated significant reduction in both fracture gap and angulation following fixation (*p* < 0.0001), with most patients achieving consolidation by 3 months, consistent with previously published data [15,16].

### 4.1. Comparison with Previous AOSF Series

This retrospective series achieved a 90% fusion rate, consistent with outcomes reported in the literature (Table 3). The single case requiring secondary posterior fusion agrees with reoperation rates of 2–10% described in the literature. Radiographically, a marked reduction in fracture gap (from 5.3 mm to 0.8 mm, *p* < 0.0001) and angulation (from 27.8° to 3.5°, *p* < 0.0005) were both within thresholds associated with favorable healing. Quantitative analyses of alignment are rarely reported, highlighting the relevance of our findings in demonstrating the corrective potential of AOSF. Apfelbaum et al. [16] emphasized anatomical reduction as the most critical determinant of fusion, while Platzer et al. [15] quantified radiographic thresholds predictive of non-union. Our findings support these conclusions: the only patient with persistent non-union presented a larger postoperative gap (3.7 mm), underlining the prognostic value of achieving <2 mm gap and <5–10° residual angulation.

Importantly, although the median age in our series was 76 years, the high fusion rate demonstrates that accurate reduction and fracture alignment can mitigate the negative impact of advanced age and reduced bone quality. The presence of a 17-year-old patient lowered the mean age value, a factor acknowledged in interpreting age-related findings.

### 4.2. Radiographic Outcomes and Predictors of Fusion

The role of fracture reduction is strongly emphasized across several studies. Apfelbaum et al. (2000) [16] showed that anatomic realignment was a key factor for achieving consolidation, while Platzer et al. (2007) [15] quantified the risk thresholds for non-union. Our findings reinforce these observations: all cases achieved near-anatomic alignment postoperatively, with residual angulation < 5° in most patients. The only patient who developed non-union presented with a larger residual gap (3.7 mm), underlying the prognostic importance of radiographic correction.

The reduction in angulation from nearly 28° to 3.5° in our cohort is comparable to the improvements reported by Dailey et al. (2010) [17], who also promote the importance of achieving sagittal alignment to reduce mechanical stress at the fracture interface. Similarly, Müller et al. (1999) [18] underlined that unsatisfactory reduction was the main cause of delayed or absent fusion, more so than patient-related factors such as age.

Postoperative immobilization strategies remain a matter of interest across published series. Some authors report satisfactory outcomes without any immobilization, others prefer semi-rigid braces [16], while several groups recommend rigid orthoses [16,19]. While our study focused on radiographic outcomes, clinical recovery is closely tied to the preservation of C1–C2 rotation and restoration of axial stability. Future prospective studies should integrate validated clinical scales (VAS, NDI, JOA) and quality-of-life measures to assess the functional benefits of AOSF more comprehensively.

**Table 3:** Clinical series and outcomes of anterior odontoid screw fixation reported in the literature.

Author	Treatment Modality	N. of Screws (%)	Sample Size (n)	Type of Study	Follow-Up (Months)	Fusion Rate (%)	Complication Rate (%)
Apfelbaum et al. (2000) [16]	Anterior odontoid screw fixation	1 (20), 2 (80)	147	Retrospective study	12	88	12
Borm et al. (2003) [20]	Anterior odontoid screw fixation	2 (100)	27	Retrospective study	6–36	74	14
Collins et al. (2008) [21]	Anterior odontoid screw fixation	1 (100)	15	Retrospective study	18	77	0
Dailey et al. (2010) [17]	Anterior odontoid screw fixation	1 (37), 2 (63)	57	Retrospective study	15	81	10
Platzer et al. (2007) [15]	Anterior odontoid screw fixation	2 (100)	110	Retrospective study	24	92	11
Müller et al. (1999) [18]	Anterior odontoid screw fixation	Not mentioned	21	Retrospective study	47	83	38
Fountas et al. (2005) [19]	Anterior odontoid screw fixation	1 (63), 2 (37)	38	Retrospective study	58	87	3
Elsaghir et al. (2000) [22]	Anterior odontoid screw fixation	2 (100)	30	Case series	25	100	0
Eap et al. (2009) [23]	Anterior odontoid screw fixation	1 (100)	36	Retrospective study	36	95	0
Yuan et al. (2018) [24]	Anterior odontoid screw fixation	1 (100)	11	Retrospective study	24	91	0

### 4.3. Comparison with Posterior Fixation Techniques and Conservative Treatment

Multiple comparative studies have demonstrated that posterior C1–C2 fusion generally achieves higher fusion rates (>95%) than AOSF, sacrificing atlantoaxial rotation and with higher surgical morbidity. A comparative overview of anterior fixation, posterior C1–C2 fusion, and conservative management is provided in Table 4.

Anterior odontoid screw fixation (AOSF) preserves motion while providing satisfactory fusion in appropriately selected patients. The two techniques differ conceptually: AOSF requires direct anatomic reduction and compression at the fracture site, in contrast posterior fixation achieves stability through rigid immobilization, regardless of gap closure. The significant radiographic correction observed in our series underlines the ability of AOSF to restore alignment, a factor likely contributing to the high union rate.

Recent literature further refines this comparison. Texakalidis et al. (2023, 2025) [6,7] demonstrated that posterior fixation techniques such as C1 lateral mass–C2 pars/pedicle and transarticular screws achieve slightly higher fusion rates, especially in osteoporotic bone or in the presence of comminution, whereas AOSF remains preferable in younger, high-demand patients with reducible Grauer IIA/IIB fractures. Gouzoulis et al. (2024) [5] reported a trend toward individualized treatment algorithms based on fracture orientation, bone quality, and patient-specific risk factors.

Additionally, novel minimally invasive and endoscopic-assisted anterior techniques may further expand the indications of AOSF in selected cases [8]. In this regard, recent percutaneous adaptations of anterior odontoid screw fixation have been proposed to simplify exposure and reduce soft-tissue dissection, while preserving screw trajectory accuracy and interfragmentary compression at the fracture site. Early institutional experiences have confirmed this technique to be safe, reproducible, and technically straightforward, particularly in elderly or frail patients where limited dissection is preferable [9]. Owing to its technical simplicity and reliance on standard fluoroscopic guidance, the percutaneous approach may represent a practical alternative in smaller or low-volume centers, where access to advanced navigation systems or specialized instrumentation is limited. When applied in carefully selected reducible fractures, it could broaden the applicability of motion-preserving fixation while maintaining procedural safety and efficiency.

Conservative management, once widely used, is now less advised, since has consistently shown lower fusion percentage (reported at 43–60%) [11] and higher rates of complications in the elderly population. Andersson et al. (2000) reported only 43% union with halo vest immobilization, predominantly due to persistent fracture displacement [30]. It should also be noted that high fusion rates with orthoses are generally limited to fractures with <2 mm displacement and <6° angulation [31], which explains the wide range of results reported in the literature.

Accordingly, AOSF represents an intermediate solution, offering biomechanical stability while preserving cervical motion.

In this context, the 90% fusion rate and marked radiographic improvement observed in our series further highlight the superiority of surgical fixation over conservative management in selected cases.

### 4.4. Technical Considerations and Risk Factors

The success of AOSF is highly dependent on patient selection. Important inclusion criteria comprise fracture angulation ≤ 60°, minimal or absent comminution, and radiographically confirmed reducibility. Several studies have shown that fracture orientation and patient bone quality are critical predictors of fusion [3,15].

Intraoperative factors such as screw trajectory, reduction maintenance, and anterior exposure also contribute significantly to the outcome. The literature review and our series both highlight the low incidence of neurovascular complications. Apfelbaum et al. (2000) and Platzer et al. (2007) both emphasized fracture line orientation and reducibility as key factors for an optimal outcome [15,16]. Our findings confirm that when these specific parameters are fulfilled, AOSF guarantees a satisfactory alignment and consolidation with minimal complications.

### 4.5. Timing of Surgery and Fusion Probability

The interval between injury and surgical fixation has also been recognized as a critical determinant of bone union. Recent multicenter data confirmed that earlier surgical intervention—typically within 7 to 10 days—significantly improves fusion rates, regardless of patient age [10]. These findings support the concept of early anatomical reduction and stabilization whenever clinically feasible. In our series, restricting inclusion to patients operated within 14 days was consistent with these observations and likely contributed to the high rate of bone consolidation obtained.

### 4.6. Strengths and Limitations

This study has several limitations. First, the small sample size limits statistical power and precludes multivariable analysis of predictors. Second, the retrospective design and lack of validated clinical outcome measures restrict conclusions on functional recovery and quality of life. Third, the absence of dynamic follow-up beyond 36 months prevents assessment of adjacent-segment degeneration or long-term functional stability. Finally, our findings are derived from a single center and a single surgeon’s experience, which may limit generalizability.

Despite these limitations, the integration of detailed radiographic analysis and standardized measurement protocols strengthens the study and provides useful insights into the technical and anatomical determinants of successful fusion.

### 4.7. Clinical Implications and Perspectives

The data presented support anterior odontoid screw fixation (AOSF) as a safe and effective treatment for type II odontoid fractures, with high fusion rates attributable to improved fracture alignment.

This technique provides a viable compromise between rigid fixation and preservation of C1–C2 motion. These findings are relevant for surgical decision-making. Radiographic reduction—particularly achieving a postoperative gap < 2 mm and angulation < 5–10°—appears to be a key determinant for the successful union, including in older patients.

Future prospective studies are needed to directly compare AOSF with posterior fusion techniques across specific subpopulations, particularly elderly patients, those with osteoporotic bone, and fractures with delayed presentation.

## 5. Conclusions

Odontoid fractures remain a major challenge in cervical spine trauma due to their biomechanical complexity and risk of pseudoarthrosis. Precise clinical and radiological assessment with CT and MRI along with classification systems such as Anderson–D’Alonzo and Grauer, is of great importance to guide treatment selection.

Management strategies range from external immobilization to anterior odontoid screw fixation and posterior C1–C2 fusion. Conservative approaches are associated with higher rates of non-union and morbidity; posterior fusion provides the most reliable stability and fusion rates, though at the cost of atlantoaxial mobility. Anterior screw fixation, when applied in selected patients with favorably oriented fractures, offers an optimal and effective balance between stability and motion preservation.

Achieving a postoperative fracture gap < 2 mm and a residual angulation < 5–10° appears to be a key radiographic predictor of bone union, even in elderly patients, and should be considered when evaluating surgical reduction. Moreover, careful patient selection remains fundamental: posterior techniques may be preferable in the presence of osteoporosis, comminution, or irreducible displacement, whereas anterior fixation is best suited for reducible Grauer IIA/IIB fractures in patients with preserved bone quality and high functional demands.

Current evidence also indicates that timely surgical intervention—ideally within 10 to 14 days from injury—significantly enhances fusion rates after AOSF [10]. In parallel, recent percutaneous minimally invasive adaptations have demonstrated that simplified exposure can reduce access-related morbidity while maintaining biomechanical stability [9].

Despite technical refinements and improved safety with modern imaging and navigation, significant challenges persist in elderly and osteoporotic populations. Optimal management therefore requires a multidisciplinary approach integrating neurosurgical, orthopedic, and radiological expertise. Future prospective multicenter studies with long-term follow-up are needed to refine patient selection, validate prognostic radiographic thresholds, and establish standardized treatment algorithms aimed at improving outcomes and quality of life.

## Figures and Tables

**Table 1:** Demographic and injury characteristics of the study population.

No.	Sex	Age at Surgery	Type of Fracture	Displacement	Angulation (°)	Associated Fracture	Trauma Mechanism
1	M	68	II B	2 mm	33	Nasal bone	Fall
2	M	66	II B	4.1 mm	14	Humerus	Fall
3	M	17	II B	4.3 mm	41	None	Motorway Accident
4	M	76	II B	10 mm	35	None	Fall
5	F	79	II B	6 mm	49	None	Fall
6	F	77	II B	6.7 mm	27	None	Fall
7	F	70	II B	4 mm	14	None	Fall
8	F	78	II A	4.6 mm	37	Radius	Fall
9	M	85	II B	7 mm	15	None	Fall
10	F	83	II B	4 mm	13	None	Fall

**Table 2:** Comparison of preoperative and postoperative displacement and angulation.

Case	Pre-Operative Displacement (mm)	Post-Operative Displacement (mm)	Pre-Operative Angle (°)	Post-Operative Angle (°)
1	2	0	33	0
2	4.1	0	14	0
3	4.3	0	41	0
4	10	3.7	35	18
5	6	0	49	0
6	6.7	1.4	27	5
7	4	0	14	0
8	4.6	0	37	0
9	7	2.6	15	12
10	4	0	13	0

**Table 4:** Comparison of treatment options for odontoid fractures.

Aspect	AOSF	Posterior C1–C2 Fusion	Conservative (Halo/Collar)
Technique	Anterior screw; needs anatomic reduction, favorable line [25,26]	Posterior screws/rods; fusion required [25]	Halo vest/collar; external immobilization [25]
Motion	Preserved [27]	Lost (~50% rotation) [27]	Preserved (if union/fibrous union) [28]
Fusion rate	~75–90% [27]	~90–100% [27]	~40–70% [28]
Complications	Nonunion, screw failure, dysphagia, esophageal injury [26]	Infection, VA injury, C2 neuralgia, motion loss [26]	Pin infection, ulcers, pneumonia, deconditioning [25]
Less suitable	Elderly, osteoporosis, comminution, posterior tilt [26]	Young/motion-demanding, VA anomalies [25]	Elderly, unstable/displaced, noncompliant [25]
Pros	Motion preserved, early mobilization [27]	Highest union/stability, versatile [27]	Non-surgical, motion preserved [25]
Cons	Strict indications, lower success in elderly [26,27]	Invasive, permanent motion loss [25,29]	Low union, high halo morbidity [25,28]

## Data Availability

The data presented in this study are available on reasonable request from the corresponding author. The data are not publicly available due to privacy and ethical restrictions.

## Abbreviations

The following abbreviations are used in this manuscript:

AOSF	Anterior odontoid screw fixation
CI	Confidence Interval
CT	Computed Tomography
MRI	Magnetic Resonance Imaging
Q-Q plot	Quantile–Quantile plot
SD	Standard Deviation
VA	Vertebral Artery
